# Detection of reassortant avian influenza A (H11N9) virus in environmental samples from live poultry markets in China

**DOI:** 10.1186/s40249-016-0149-2

**Published:** 2016-06-08

**Authors:** Ye Zhang, Shu-Mei Zou, Xiao-Dan Li, Li-Bo Dong, Hong Bo, Rong-Bao Gao, Da-Yan Wang, Yue-Long Shu

**Affiliations:** National Institute for Viral Disease Control and Prevention, Chinese Center for Disease Control and Prevention, Beijing, 102206 China; Key Laboratory for Medical Virology, National Health and Family Planning Commission, Beijing, 102206 China

**Keywords:** Avian influenza, H11N9, Genetic characterization, Reassortant

## Abstract

**Background:**

Avian influenza viruses have caused human infection and posed the pandemic potential. Live poultry markets are considered as a source of human infection with avian influenza viruses. Avian influenza routine surveillance of live poultry markets is taken annually in China. We isolated the 2 H11N9 influenza virus from the surveillance program. To better understand the risk caused by these new viruses, we characterize the genetic and pathogenicity of the two viruses.

**Methods:**

Viral isolation was conducted with specific pathogen-free (SPF) embryonated chicken eggs. Whole genome was sequenced, and phylogenetic analysis was conducted.

**Results:**

Two H11N9 viruses were identified, with all 8 segments belonging to the Eurasian lineage. The HA, NA, M, NS and PA genes were similar to virus isolates from ducks, and the NP, PB2 and PB1 gene segments were most similar to those viruses from wild birds, indicating that the H11N9 viruses might represent reassortant viruses from poultry and wild birds. The HA receptor binding preference was avian-like, and the cleavage site sequence of HA showed low pathogenic. The NA gene showed 94.6 % identity with the novel H7N9 virus that emerged in 2013. There was no drug resistance mutation in the M2 protein. The Asn30Asp and Thr215Ala substitutions in the M1 protein implied a potentially increased pathogenicity in mice. Both viruses were low-pathogenic strains, as assessed by the standards of intravenous pathogenicity index (IVPI) tests.

**Conclusion:**

Two reassortant H11N9 avian influenza viruses were detected. These viruses showed low pathogenicity to chickens in the IVPI test. Public health concern caused by the reassortant H11N9 viruses should be emphasized during the future surveillance.

**Electronic supplementary material:**

The online version of this article (doi:10.1186/s40249-016-0149-2) contains supplementary material, which is available to authorized users.

## Multilingual abstracts

Please see Additional file 1 for translations of the abstract into the six official working languages of the United Nations

## Background

Wild waterfowl are considered to be the natural reservoir of influenza A virus. There are currently 16 HA subtypes (H1-H16) and nine NA subtypes (N1-N9) circulating in birds [1], and the H17N10 and H18N11 subtypes have been detected in bats [2]. The 16 avian HA subtypes have been detected in North America and Europe; among them, H4, H6 and H9 are the most common subtypes, followed by H3, H7, H11 and H5 in birds and poultry [3, 4]. By 2008, at least nine avian influenza HA subtypes and six NA subtypes had been reported in domestic ducks in eastern China [5].

Since 1900, H1, H2 and H3-subtype influenza A viruses have infected humans and have circulated within the human population. The first human infection with a highly pathogenic avian influenza H5N1 virus occurred in Hong Kong in 1997 [6]. Subsequently, human infections with other subtypes, such as H9N2, H7N2 and H7N3, were occasionally reported. In 2013, human infection with novel influenza A (H7N9) virus was first identified in China [7]. Subsequently, the first human infections with an H6N1 virus and an H10N8 virus were reported in Taiwan and Jiangxi, China, respectively [8, 9].

Epidemiological investigation has shown that live poultry markets (LPMs) are important sources of human infection with avian influenza viruses [10]. Avian influenza viruses can be detected in poultry as well as in environmental samples from LPMs [11]. The contaminated environments may provide continual sources to the new coming batches of poultry.

Since 2009, we have undertaken a nationwide surveillance program to monitor avian influenza viruses in poultry-related environmental samples. In this study, we isolated two reassortant H11N9 viruses from an LPM in Jiangxi province during routine surveillance in 2009. Genetic and molecular evolutionary analyses indicate that they are low-pathogenic avian influenza viruses. To our knowledge, this is the first report to identify a reassortant avian H11N9 virus in environmental samples from an LPM.

## Methods

### Sample collection

Our nationwide routine avian influenza virus surveillance of environmental samples has been conducted in China since January 2009. The samples, including bird feces, drinking or contaminated water, and environmental swabs, were collected monthly from LPMs, slaughterhouses and farms. From January 2009 to December 2013, 33 049 samples were collected from 142 cities in 31 provinces. The samples were placed into 15 mL tubes containing viral transport medium and sent to the laboratory for further testing. The viral transport medium consisted of 4 mL of minimum essential medium containing 0.5 % BSA, 10 % glycerol, 2 × 10^6^ U/L penicillin G, 200 mg/L streptomycin, 2 × 10^6^ U/L polymyxin B sulfate, 250 mg/L gentamicin, 60 mg/L ofloxacin-HCl, 0.2 g/L sulfamethoxazole and 5 × 10^5^ U/L nystatin (Sigma, St. Louis, MO, USA).

### RNA extraction and real-time PCR

An RNeasy Kit (Qiagen, Chatsworth, CA, USA) was used to extract viral RNA from the samples. Influenza A virus RNA was detected by real-time PCR (AgPath; Applied Biosystems, Foster City, CA, USA) targeting a matrix gene on a Stratagene Mx3005P thermocycler. The real-time RT-PCR amplification protocol included steps of 45 °C for 10 min and 95 °C for 10 min and then 40 cycles of 95 °C for 15 s and 60 °C for 45 s.

### Viral isolation

Virus-positive samples were shipped to the Chinese National Influenza Center for viral isolation. The samples were inoculated into the allantoic cavities of 9-day-old embryonated chicken eggs that were then incubated at 37 °C for 48 h before being chilled at 4 °C overnight. The allantoic fluid was harvested, and virus identification was performed with a hemagglutination assay with 0.5 % turkey red blood cells.

### Gene sequencing

Eight gene segments of each virus were amplified with a One-Step RT-PCR kit (QIAGEN, Germany) according to the manufacturer’s protocol. Complete genome amplification was performed using specific primers [12]. PCR products were purified with a QIAquick Gel Extraction Kit (QIAGEN, Germany) according to the manufacturer’s protocol. Sequencing was performed using a BigDye Terminator v3.1 Cycle Sequencing Kit on an ABI PRISM 3700 xl DNA Analyzer (Applied Biosystems) according to the manufacturer’s protocol.

### Phylogenetic analysis

Sequences were assembled and edited with Lasergene 8.1 (DNASTAR). Neighbor-joining (NJ) trees were constructed using MEGA 5.0. The bootstrap value was tested with 1000 replications for each gene segment. Homology analyses of nucleic acids and amino acids were performed on the NCBI website with BLAST. All of the reference sequences used in the phylogenetic comparison were obtained from GenBank.

### IVPI test

Fresh infective allantoic fluid with a HA titer > 16 was diluted 1:10 in sterile isotonic saline. A sample (0.1 ml) of the diluted virus was injected intravenously into each of 10 6-week-old SPF chickens. All of the chickens were examined daily for 10 days and scored based on the condition of each chicken: 0 (normal), 1 (sick), 2 (severely sick) and 3 (dead), as described in the OIE/WHO guidelines [13].

## Results

### Molecular detection, viral isolation and subtype identification

The proportion of influenza M gene-positive environmental samples was 7.9 %, and the positive viral isolation rate was 1.5 %. The H2, H3, H4, H5, H6, H7, H9 and H11 subtypes were detected between year 2009 and 2013. Among all of the viral isolates, 2 H11N9 avian influenza viruses were separately isolated from two LPMs in Wuning city, Jiangxi province. Both viruses were isolated in 2009 and were named A/environment/Jiangxi/26/2009(H11N9) (JX/26/2009) and A/environment/Jiangxi/28/2009(H11N9) (JX/28/2009). JX/26/2009 was collected from Xinning LPM on July 8, 2009, and JX/28/2009 was isolated from Guai LPM on July 8, 2009. The complete genome sequences of the influenza viruses have been deposited in GenBank under accession numbers KC881287 to KC881302.

### Homology comparison

JX/26/2009 and JX/28/2009 were highly homologous, with greater than 99 % identity in the nucleotide sequences of each segment. The lengths of the PB2, PB1, PA, HA, NA, NP, M and NS genes were 2341, 2341, 2233, 1754, 1453, 1565, 1027 and 890 bp, respectively. The HA gene showed the highest identity (99 %) with the Eurasian A/duck/Chiba/11/2007(H11N9) virus, whereas the NA gene sequence showed 99 % identity with the Eurasian A/duck/Vietnam/G30/2008(H11N9) virus. The NA genes of the viruses were 94.6 % identical to A/Anhui/1/2013(H7N9), the novel A(H7N9) virus identified in March 2013 in China. The nucleotide sequences of the internal genes showed high similarity with other avian influenza viruses from wild birds or ducks. The viruses in GenBank that had the highest amino-acid sequence homology to our newly identified viruses are shown in Table 1.

**Table 1:** Comparisons of A/Environment/Jiangxi/26/2009(H11N9) with Isolates in GenBank with highest nucleotide and amino acid identity

Gene segment	Virus with the highest nucleic acid homology	Percent homology (%)	GenBank ID
PB2	A/velvet A/scoter/Mongolia/883 V/2009(H4N6)	99	KC986346.1
PB1	A/mallard/Korea/GH170/2007(H7N7)	99	FJ750870.1
PA	A/duck/Jiangxi/25186/2009(H7N6)	98	KF260253.1
HA	A/duck/Chiba/11/2007(H11N9)	99	AB669139.1
NP	A/northern pintail/Hong Kong/MPC2085/2007(H11N9)	98	KF259789.1
NA	A/duck/Vietnam/G30/2008(H11N9)	99	AB593465.1
M	A/duck/Jiangxi/16326/2010(H7N7)	99	KF259249.1
NS1	A/duck/Nanjing/1102/2010(H4N6)	99	KC683707.1

### Phylogenetic analysis

**Fig. 1:**
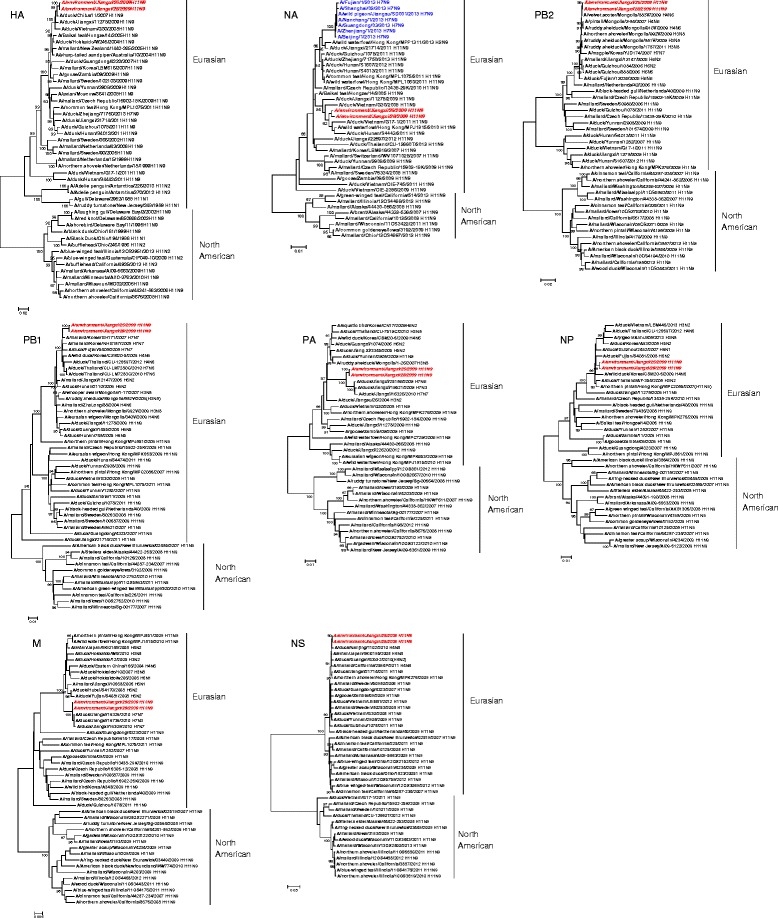
Phylogenetic analysis of the A/Environment/Jiangxi/26/2009 and A/Environment/Jiangxi/28/2009 viruses. A/Environment/Jiangxi/26/2009 and A/Environment/Jiangxi/28/2009 are indicated by bold type. Numbers below nodes represent bootstrap values from 1000 replicates

The results of a phylogenetic analysis showed that the eight gene segments of the 2 H11N9 viruses isolated in this study all belonged to the Eurasian lineage, which was distinct from the branch of North American lineages in the tree (Fig. 1). The HA genes were closely related to A/duck/Chiba/11/2007(H11N9), whereas their NA genes were in a separate subgroup from the A(H7N9) influenza virus.

The PB2, PB1, PA, NP, M and NS genes clustered with A/Velvet scoter/883 V/2009(H4N6), A/Mallard/Korea/GH171/2007(H7N9), A/Duck/Jiangxi/25186/2009(H7N6), A/Wild duck/Korea/CSM20-5/2009(H4N6), A/Duck/Jiangxi/26326/2010(H7N7) and A/Duck/Nanjing/1102/2010(H4N8), respectively.

### Molecular analysi*s*

The HA cleavage sites of the 2 H11N9 viruses were AIASR/G, which is characteristic of low-pathogenic avian influenza viruses. The Q226 residue of HA indicated that the receptor binding sites still recognize the α-2, 3 receptor (in H3 numbering). The mammalian-adaptation mutations of PB2 (627 K and 701 N) were not found in either of the viruses, indicating that the viruses originated from an avian source.

The N30D and T215A substitutions in the M1 protein, which have been associated with increased H5N1 pathogenicity in mice [14], were found in JX/26/2009 and JX/28/2009. No adamantine-resistance or neuraminidase drug-resistance mutations were detected in the M2 or NA proteins, respectively. The main amino acid mutations are shown in Table 2.

**Table 2:** Key amino acid residues analysis of A/environment/Jiangxi/26/2009(H11N9) and A/environment/Jiangxi/28/2009(H11N9)

Protein	Mutation	Function	JX/26/2009 and JX/28/2009
HA	Q226L	Receptor biding site. Q is the avian signature.	Q
PB2	E627K	Enhances pathogenicity in mice	E
	D701N		D
M1	N30D	increase the virulence of H5N1 avian influenza viruses in mice	D
	T215A		A
M2	S31N	resist to amantadine	S
	H274Y		H
NA	E119V	resist to oseltamivir or zanamivir	E
	R152K		R

### IVPI test

The IVPI scores were calculated according to the OIE/WHO guidelines [13]. The IVPI scores of JX/26/2009 and JX/28/2009 were 0.43 and 0.6, respectively, indicating that these 2 H11N9 viruses are low-pathogenic avian influenza viruses.

## Discussion

H11N9 has been reported in wild waterfowl in Brazil, Belgium, the Republic of Kazakhstan, and Japan [15–19]. This subtype virus has also been isolated from ducks in southern China and Hong Kong SAR [20].

From 2009 to 2013, two H11N9 avian influenza viruses were isolated during routine surveillance. The samples were collected from chicken cages in LPMs. Phylogenetic analyses indicated that all the gene segments belonged to the Eurasian lineage. The HA, NA and NP genes originated from H11N9 viruses, whereas the internal gene segments clustered with H7N7, H7N6 and H4N6. The Eurasian-lineage HA gene clusters with recently reported H11N9 viruses that have been found in wild birds or ducks (Fig. 1). H11N9 viruses have been identified as the donors of the NA gene of H7N9 virus in the 2013 [21]. The NA phylogenetic trees showed that the two H11N9 viruses in this study were in the Eurasian lineage together with NA of the H7N9 viruses, though they were in a separate subgroup from the H7N9 viruses. The HA, NA, M and NS genes of the two strains of H11N9 viruses were derived from domestic ducks, whereas the polymerase and NP genes were derived from wild birds. It has been suggested that shared water sources between waterfowl and poultry may enhance the possibility of reassortant avian influenza virus occurrence [22]. Indeed, the H11N9 viruses in our report were collected in water-rich areas in Jiangxi province. We hypothesize that the virus originated via the occasional reassortment of viral gene segments from wild birds, waterfowl and poultry. Recently, an H11N9 virus has been isolated from a domestic duck in LPMs in eastern China; this virus obtained its genes from H11, H3, H10 and H7 avian influenza viruses from ducks in Chinese LPMs [23]. Because LPMs provide favourable conditions for the reassortment of avian influenza viruses, this is important to strengthen the surveillance in LPMs to monitor the distribution and genetic variation of avian influenza viruses.

Among influenza A viruses, H5 and H7 are considered to be notable avian influenza viruses, but not all H5 and H7 viruses display high pathogenic. Low-pathogenic avian influenza viruses can be precursors of highly pathogenic viruses [24, 25]. The two H11N9 viruses in this study are low-pathogenic avian influenza viruses. Their receptor binding sites retained an avian virus-like character. The N30D and T215A mutations observed in the M1 protein might increase the pathogenicity of these viruses in mice.

Reassortment among different influenza viruses is considered to be the main mechanism for the generation of new viruses, which may bear enhanced mammalian transmission and pandemic potential. It has been reported that H11N9 viruses can be transmitted directly to hunters from ducks [26]. New H11-subtype viruses have also been isolated from penguins [27]. Therefore, influenza virus subtypes other than the dominant H5, H7 and H9 subtypes should be emphasized. Continual monitoring is especially important for reassortant viruses, which may possess new characteristics that have implications for public health.

## Conclusion

Two H11N9 influenza viruses were isolated from the live poultry markets in Jiangxi during the routine surveillance. All the segments of the viruses were belonging to the Eurasian lineage of influenza virus genes. The segments of the viruses are originated from both poultry and wild birds. The character of the genes origination highlights the important of Jiangxi province as the possible hot place of mixture the influenza viruses from both migratory wild birds and poultry. The NA genes of the two viruses are different from the novel H7N9 virus of 2013. The Both viruses were low-pathogenic strains from the IVPI tests and the molecular genes markers. The reassortant virus may possess new characteristics that may be of concern to public health, and continuous surveillance should be emphasized.

## Abbreviations

IVPI, intravenous pathogenicity index; LPM, live poultry market; r RT-PCR, real-time reverse transcription-polymerase chain reaction; RT-PCR, reverse transcription-polymerase chain reaction; SPF, specific pathogen free

## Additional file

Additional file 1: Multilingual abstracts in the six official working languages of the United Nations. (PDF 444 kb)
