# Supplementary material for: Detection of reassortant avian influenza A (H11N9) virus in environmental samples from live poultry markets in China
Source: Infect Dis Poverty. 2016 Jun 8;5:59. doi: 10.1186/s40249-016-0149-2 (PMC4897819; doi:10.1186/s40249-016-0149-2)
Supplement: Additional file 1: — Multilingual abstracts in the six official working languages of the United Nations. (PDF 444 kb) [file 40249_2016_149_MOESM1_ESM.pdf]

Translation of the abstract into the five official working languages of the United Nations

الكشف عن اندماج تضاعفي لفيروس أنفلونزا الطيور A (H11N9) في عينات بيئية من أسواق الدواجن الحية في الصين

يي تشانغ، شو مي زو، شياو- دان لى، لى بو دونغ، هونغ بو ، رونغ- باو غاو، دا- يان وانغ، يو- لونغ شو

الملخص

الخلفية: للتمييز وراثياً تم عزل 2 فيروس إنفلونزا H11N9 من مسحات قفص دجاج في سوق لبيع الطيور الحية في مقاطعة جيانغشي، الصين بواسطة الترصد الروتيني.

الطرق: أجريت العزلة الفيروسية من بيض دجاج يحتوي على جنين خالٍ من أى مرض. وتم تسلسل ثمانية قطاعات جينية، وإجراء تحليل النشوء والتطور.

النتائج: تم تحديد اثنين من فيروسات H11N9 ، مع انتماء جميع الشرائح الثمانية إلى سلالة الأوراسي. وكانت جينات HA ، NA، M، NS، PA مشابهة للجينات من البط، وكانت فئات الجينات NP ، PB2 PB1 هي الأكثر تشابهاً لتلك الموجودة في الفيروسات من الطيور البرية، وأشارت إلى أن فيروسات H11N9 قد تمثل فيروسات الاندماج التضاعفي من الدواجن والطيور البرية. كان التفضيل الرابط للمُسْتَقْبِل HA مثل الطيور ، وأظهر تسلسل موقع الانشقاق انخفاضاً مَرَضِّياً. وأظهر هذا الجين NA 94.6٪ تماثلاً مع فيروس H7N9 الجديد الذي ظهر في عام 2013. لم يكن هناك طفرة مقاومة العقار في البروتين M2 . ألمحت بدائل Asn30Asp و Thr215Ala في البروتين M1 إمكانية لزيادة المرضية في الفئران. وكان كلا الفيروسيين سلالات منخفضة المرضية ، وفقاً لتقييم معايير اختبارات مؤشر المرضية عن طريق الوريد.(IVPI)

الخلاصة: تم الكشف عن اثنين اندماج تضاعفي لفيروسات إنفلونزا الطيورH11N9 . وأظهرت هذه الفيروسات أقل مرضية للدجاج في اختبار IVPI . قد يمتلك فيروس الاندماج التضاعفي خصائصاً جديدة قد تثير قلقاً على الصحة العامة، وينبغي التأكيد على المراقبة المستمرة.

Translated from English version into Arabic by Free bird, through

TRANSLATORS
WITHOUT BORDERS

中国活禽市场环境样本中重组 H11N9 禽流感病毒的检测分析

张烨，邹淑梅，李晓丹，董丽波，薄洪，高荣保，王大燕，舒跃龙

摘要

引言： 通过禽流感常规监测得到两株H11N9禽流感病毒，两株H11N9病毒从中国江西省活禽市场的鸡笼涂抹拭子中分离并进行病毒基因特性分析。

方法： 通过接种无特定病原体鸡胚进行病毒分离、 测定病毒的8个基因片段序列并进行基因进化分析。

结果： 两株H11N9病毒的8个基因片段均属于欧亚谱系。 HA, NA, M, NS 和PA 基因序列与鸭中病毒的相应基因最为相似。NP,PB2,PB1的基因序列与野鸟中病毒相应基因最为接近。基因进化分析表明H11N9 病毒的基因是由家禽和野鸟流感病毒之间重配产生。HA受体结合特征分析表明其为禽源受体结合且裂解位点氨基酸序列表明该病毒为低致病性禽流感病毒。NA基因与2013年出现的H7N9病毒NA核苷酸序列相似性达94.6%。M2 蛋白序列分析表明该病毒没有耐药性位点的突变。M1 蛋白存在Asn30Asp和 Thr215Ala突变，具有对小鼠潜在的致病性。静脉致病指数测定显示两株H11N9病毒均为低致病性禽流感病毒。

结论： 本研究分离出两株重组的 H11N9 禽流感病毒。通过静脉致病指数实验测定两株 H11N9 病毒均对禽是低致病性。研究结果表明加强禽流感病毒的监测，及时发现重配病毒对禽流感防控具有重要意义。

Translated from English version into Chinese by Zhang Ye.

Détection de Virus A (H11N9) Réassortis de la Grippe Aviaire dans des prélèvements environnementaux sur des Marchés de la Volaille en Chine

Ye Zhang, Shu-Mei Zou, Xiao-Dan Li, Li-Bo Dong, Hong Bo, Rong-Bao Gao, Da-Yan Wang, Yue-Long Shu

Résumé

Contexte: Pour caractériser génétiquement les virus 2 H11N9 de la grippe aviaire, isolés par des prélèvements dans des cages à poule sur un marché de volailles vivantes de la province de Jiangxi, en Chine, par une surveillance de routine.

Méthodes: l'isolement viral a été réalisé sur des œufs embryonnés exempts d'organismes pathogènes spécifiques (EOPS). Huit segments de gènes ont été séquencés, et l'analyse phylogénétique a été réalisée.

Résultats: deux virus H11N9 ont été identifiés, les 8 segments appartenant à la lignée eurasienne. Les gènes HA, NA, M, NS et PA étaient similaires à des gènes de canards, et les segments de gènes NP, PB2 et PB1 étaient très similaires à ceux identifiés dans des virus provenant d'oiseaux sauvages, indiquant que les virus H11N9 pourraient représenter les virus réassortis provenant de volailles et d'oiseaux sauvages. La préférence du récepteur HA était aviaire, et la séquence au niveau de la zone de clivage a montré une faible pathogénicité. Le gène NA a montré une identité de 94.6% avec le nouveau virus H7N9 apparu en 2013. Il n'y avait pas d'évidence de mutation de résistance médicamenteuse dans la protéine M2. Les substitutions d'Asn30Asp et Thr215Ala dans la protéine M1 ont laissé entendre un potentiel de pathogénicité accrue chez les souris. Les deux virus étaient de souches à faible pathogénicité, comme cela a été évalué par les normes des tests de l'indice de pathogénicité intraveineux (IVPI).

Conclusion: deux virus H11N9 réassortis de la grippe aviaire ont été détectés. Ces virus ont montré une faible pathogénicité pour les poulets au cours du test IVPI. Il se peut que le virus réassortis possède des caractéristiques pouvant susciter de la préoccupation à l'égard de la santé publique, et une surveillance constante devrait être soulignée.

Translated from English version into French by Ode Laforge, through

TRANSLATORS
WITHOUT BORDERS

Выявление реассортантного вируса птичьего гриппа A (H11N9) в природных образцах с птичьих рынков в Китае

Йе Жан, Шу-Мей Дзоу, Сяо-Дан Ли, Ли-Бо Донг, Хон Бо, Жонг-Бао Гао, Да-Ян Ванг, Юэ-Лонг Шу

Краткое содержание

Общие сведения: Для генетической экспертизы вирусов гриппа 2 H11N9 брались мазки из птичьих клеток на рынке живой птицы в провинции Цзянси (Китай), которые впоследствии подвергались обычной процедуре.

Методы: Вирус был изолирован из беспатогенных куриных яиц с зародышами. Были секвенирован восемь сегментов гена, проведен филогенетический анализ.
Результаты: Идентифицировано два вируса H11N9 со всеми 8 сегментами, принадлежащими к евразийской линии. Гены HA, NA, M, NS и PA были подобны генам уток, а сегменты NP, PB2 и PB1 были схожи с с генными сегментами вирусов диких птиц, что говорит о том, что вирусы H11N9 могут быть псевдовирионами домашней и дикой птицы. Рецепторное связывание HA было птичьеподобным, а последовательность участка расщепления свидетельствовала о низкой патогенности. Ген NA показал 94.6% идентичность с новым вирусом H7N9, который появился в 2013 году. В белке не было выявлено устойчивой к медикаментам мутации. Замены аллелей Asn30Asp и Thr215Ala в белке M1 предполагают увеличение патогенности у мышей. Оба вируса были низкопатогенными штаммами, как показали результаты анализов индекса внутривенной патогенности.
Заключение: Выявлено два реассортантных вируса птичьего гриппа H11N9. Эти вирусы являются низкопатогенными, как показали анализы индекса внутривенной патогенности у кур. Реассортантный вирус может обладать новыми особенностями, опасными для здоровья людей, необходимо продолжить наблюдение.

Translated from English version into Russian by Jekaterina Merkuljeva, through

TRANSLATORS
WITHOUT BORDERS

Detección de virus recombinante de gripe aviar A (H11N9) en muestras ambientales tomadas en mercados de aves vivas de corral en China

Ye Zhang, Shu-Mei Zou, Xiao-Dan Li, Li-Bo Dong, Hong Bo, Rong-Bao Gao, Da-Yan Wang, Yue-Long Shu

Resumen
Información de referencia: Caracterizar genéticamente dos virus de la gripe H11N9 tomados mediante frotis de jaulas de pollos en un mercado de aves vivas en la provincia de Jiangxi (China) como vigilancia rutinaria.
Métodos: El aislamiento del virus se realizó con huevos gallina embrionados exentos de patógenos específicos. Se secuenciaron ocho segmentos de genes y se llevaron a cabo análisis filogenéticos.
Resultados: Se identificaron dos virus de H11N9, con sus 8 segmentos pertenecientes al linaje euroasiático. Los genes HA, NA, M, NS y PA fueron similares a los genes de patos, y los segmentos de genes NP, PB2 y PB1 fueron los más similares a los virus de aves salvajes, indicando así que los virus H11N9 podrían representar los virus recombinantes de aves de corral y aves salvajes. La vinculación preferente del receptor HA era de tipo aviar y la secuencia en el sitio de clivaje mostró una baja patogenicidad. El gen NA mostró un nivel de identidad del 94,6% con el nuevo H7N9 surgido en 2013. No había mutación de resistencia al fármaco en la proteína M2. Las sustituciones Asn30Asp y Thr215Ala en la proteína M1 implicaban una mayor patogenicidad potencial en ratones. Ambos virus eran cepas de baja patogenicidad según lo establecido en los estándares de las pruebas de índice de patogenicidad intravenosa (intravenous pathogenicity index, IVPI).
Conclusión: Se detectaron dos virus recombinantes de gripe aviar H11N9. Estos virus mostraron una baja patogenicidad a los pollos en la prueba IVPI. El virus recombinante podría tener unas nuevas características que sean de interés para la salud pública y es preciso realizar una vigilancia continua.

Translated from English version into Spanish by Sergio Lorenzi, through

TRANSLATORS
WITHOUT BORDERS
